# Prospective Evaluation of Blood Borne Virus Testing in Custody Suites in North‐East England

**DOI:** 10.1111/jvh.70042

**Published:** 2025-06-10

**Authors:** Danielle Rayner, Francesca McCullough, Kate McQue, Kerry Jones, Caroline Allsop, Jenna Bell, Carolyn Miller, Stuart McPherson

**Affiliations:** ^1^ Liver Unit Newcastle Upon Tyne Hospitals NHS Foundation Trust Newcastle upon Tyne UK; ^2^ Mitie Care and Custody Healthcare Stockport UK; ^3^ Translational and Clinical Research Institute, Newcastle University Newcastle upon Tyne UK

**Keywords:** direct acting antiviral treatment, hepatitis C virus, injecting drug use, people who inject drugs, police, prison

## Abstract

Drug‐related crime is a common reason for arrest. Therefore, some arrested individuals are at risk of hepatitis C virus infection (HCV). We present the outcomes of a blood borne virus (BBV) testing programme in custody suites in North‐East England. Individuals reviewed in healthcare departments of three custody suites were offered dry blood spot BBV testing for HCV, hepatitis B (HBV) and human immunodeficiency virus (HIV) between July 2021 and June 2024. Data were collected prospectively on numbers tested, virology results and treatment outcomes. In total, 582 had BBV testing (508 [87%] valid HCV antibody and HCV RNA tests). Overall, 13% (64) had a detectable HCV antibody and 6% (31) had detectable HCV RNA indicating active HCV infection. Of these, 12 (39% of HCV RNA positive; 2.3% of all tested) were newly identified infections. Twenty‐four individuals (77%) commenced antiviral treatment. Six individuals did not start antiviral treatment because of non‐engagement, and one is in treatment workup. Of the 33 HCV antibody–positive, but RNA‐negative individuals, 20 (61%) had previous antiviral treatment and achieved SVR, nine (27%) were thought to have spontaneously cleared the infection and four (12%) were on treatment at the time of testing. There were no cases of HBV or HIV identified. Dry blood spot testing for BBVs in custody suites is feasible and identifies a high proportion with active HCV infection, with the majority commencing antiviral treatment. Viral hepatitis services should consider expanding BBV testing to custody suites to help work towards HCV elimination.

## 1 Introduction

Globally, there are an estimated 50 million individuals with chronic hepatitis C virus infection (HCV) and a large proportion remain undiagnosed [1]. The World Health Organization (WHO) has targeted the elimination of viral hepatitis by 2030, and some countries are on track to achieve this for HCV. However, new HCV infections and reinfections after antiviral treatment are frequent among people who inject drugs (PWID) and this may slow progress towards elimination [2]. Therefore, prompt identification and treatment of those with active HCV is essential to reduce the risk of ongoing transmission. Blood borne virus (BBV) testing programmes targeting high‐risk individuals are available in many settings, including addiction services and prisons, where universal opt‐out testing is recommended, with annual retesting recommended for those with ongoing risk [3, 4]. However, testing rates frequently fall below desirable levels in many services. Moreover, many ‘at risk’ individuals do not attend healthcare facilities regularly, so may not be offered testing at all. There is therefore a need to expand BBV testing to novel settings.

Drug‐related crime is one of the most common reasons for arrest in many countries. In England and Wales, approximately 210,000 drug‐related offences are recorded by Police each year [5]. Therefore, some individuals who are arrested are at increased risk of infection with BBVs, and offering testing in the custody suite may offer an opportunity to diagnose HCV. Here we present the outcomes of a BBV testing programme in custody suites in part of North‐East England.

## 2 Methods

The BBV testing programme was introduced to Northumbria Custody suites in July 2021, beginning in Middle Engine Lane Suite with subsequent roll out to Forth Banks and Southwick suites in August 2021. Northumbria Police serves a population of 1.5 million people and covers an area of more than 2000 square miles in the North East of England, from the Scottish border down to County Durham and from the Pennines across to the North East coast. Nurses are embedded in all three stations. All individuals aged ≥ 18 years who were referred to healthcare staff in the custody suite were eligible to be offered BBV testing except people arrested on suspicion of sexual assault or road traffic offences. Detainees' health needs are governed by Police and Criminal Evidence Act Code C, which details that medical attention should be given as soon as practical to any detainee who is deemed to be suffering from physical or mental ill health, has drug or alcohol dependence or requests medical attention [6]. Approximately 50% of detainees are reviewed by a healthcare practitioner following arrest.

Those who accepted had dry blood spot testing for BBVs by trained healthcare staff in the custody suite. Samples were analysed for HCV antibody, HCV ribonucleic acid (RNA), hepatitis B surface antigen (HBsAg) and human immunodeficiency virus (HIV) as previously described [7]. Test results were mailed to the participants within 2 weeks of testing. Individuals who tested HCV RNA, HBsAg or HIV positive were offered an appointment in the viral hepatitis clinic within 2 weeks to arrange further investigations and initiate treatment, where appropriate. Individuals with active HCV were offered treatment with direct acting antivirals (DAA) as per National Health Service (NHS) England recommendations. Sustained virological response (SVR) was defined as HCV RNA not detected, 12 weeks after completion of DAA treatment. Although individuals were tested for three BBVs, the major focus of this work was around HCV.

Data were collected on the number of individuals accepting BBV testing, test results and subsequent treatment outcomes between July 2021 and the end of June 2024 (3 years). For individuals found to be HCV positive, our regional HCV Database, HepCare, was interrogated to determine whether they were already known to have HCV or whether this was a newly identified infection. HepCare holds detailed clinical and laboratory data on all patients with HCV in our region.

This service improvement project was funded by NHS England and registered with the Newcastle upon Tyne Hospitals NHS Foundation Trust Clinical Governance Department (ref. no.: 17178).

## 3 Results

**FIGURE 1:**
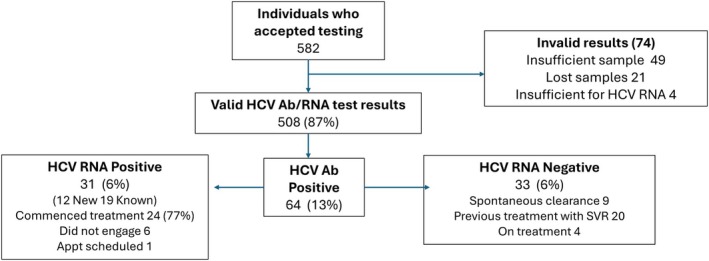
Overview of the outcomes of HCV testing in the custody suites.

The total number of arrests leading to detention across three custody suites during the project was 93,851 and it is estimated that half of those were reviewed in the healthcare unit, and some individuals were arrested more than once. Therefore, from a total of approximately 30–40,000 individuals, 582 (Figure 1) people had BBV performed. Overall, 508 (87%) had valid testing for HCV antibody and HCV RNA. In total, 13% (64) of the individuals tested had a detectable HCV antibody, including 6% (31) who had detectable HCV RNA indicating active HCV infection. Of these, 12 (39% of HCV RNA positive; 2.3% of all tested) were newly identified infections and 19 were known to have had a positive HCV test previously. Of those with active HCV, 24 (77%; 15 who were previously known HCV and nine new infections) were commenced on antiviral treatment and 12 (50%) are known to have achieved SVR at the time of writing. Six individuals did not start antiviral treatment because of non‐engagement with the treatment service, and one is currently in treatment workup.

Of those 33 individuals who were HCV antibody positive but RNA negative, 20 (61%) had previously had antiviral treatment and achieved SVR, nine (27%) were thought to have spontaneously cleared the infection, and four (12%) were on treatment at the time of testing.

There were no cases of HBV or HIV identified.

Unfortunately, 74 individuals had invalid testing; this was due to insufficient samples in 49 and lost samples in 21. The majority of invalid sampling occurred at the beginning of the project and improved following further training on the DBST technique.

## 4 Discussion

Despite there being a significant increase in the numbers of individuals with HCV being treated and cured worldwide, it remains a highly prevalent chronic infection. In England, where there has been a significant investment in an HCV elimination programme, the prevalence of HCV has fallen by more than 50% between 2015 and 2022, leaving an estimated 62,600 untreated individuals, many who remain undiagnosed [3]. Treating services are finding it harder to find the remaining cases as the majority of those identified have received treatment [4]. There therefore needs to be an expansion of HCV testing to identify the remaining infections. Given that drug‐related crime is a frequent reason for arrest and a relatively high proportion of detainees have risk factors for HCV, we introduced BBV testing to custody suites in our area. Overall, more than 500 people were tested over a 3‐year period, with 13% being HCV antibody positive and 6% having active HCV. Twelve of these infections (2.3%) were new diagnoses. Importantly, a high proportion (77%) of those with active HCV were successfully linked to care and started antiviral treatment. This is the first report of a BBV testing programme in police custody suites, and given the high yield of infections, other areas should consider introducing a similar programme.

Overall, the diagnostic yield for active HCV infection was high in custody suites compared with more general settings, and with good linkage to care, this makes this a cost‐efficient approach to identify and treat HCV. By comparison, universal opt‐out testing for HCV in an emergency department in London in 2022 yielded 0.21% with active HCV infection [8]. Universal opt‐out BBV testing at reception to Prison is now widely practised in prisons in England with an overall testing uptake of 72% [3]. A reception prison BBV testing and treatment programme have been operational since 2017 in our region with good uptake (79% in 2022), a high yield of active HCV (6.2% in 2022) and a large proportion (85%) commencing antiviral treatment. It could be argued that many of the cases of HCV identified in the custody suite would subsequently be diagnosed by prison BBV testing. However, the majority of arrested individuals are released without charge or receive non‐custodial sentences, so expanding testing to custody suites expands the opportunity to test in the penal system, potentially identifying cases that would otherwise not have been identified.

Of the 19 individuals who had a previous diagnosis of HCV and were untreated, 15 went on to engage with treatment after their custody suite test. This reinforces the utility of screening in custody suites to re‐engage individuals in HCV treatment services as well as diagnose new cases.

Introducing BBV testing programmes in new environments does present challenges. Firstly, 13% of the samples were invalid in the programme, mainly due to insufficient sample for reliable analysis. This predominantly occurred at the beginning of the project and highlights the need for good training and regular review of the programme with ‘top up’ training where required. There were also practical difficulties contacting individuals with test results due to some individuals providing incorrect addresses or telephone numbers and others being homeless. Despite these challenges, linkage to antiviral treatment was good.

Overall, the total proportion of people tested out of those potentially eligible was relatively low. This was due to a variety of reasons, including the service being very busy at times, some individuals not being appropriate to offer tests to due to intoxication or significant mental health deterioration, and some individuals who declined as testing was a low priority for them. The relatively high prevalence of HCV in the cohort also suggests there was likely to be a selection bias, with those at higher risk of HCV more likely to be offered or accept a test. Although no cases of HBV or HIV were found during this project, custody suites in regions with a higher prevalence of HBV and HIV may diagnose these infections, and further work should be conducted on this approach in other areas.

## 5 Conclusion

Dry blood spot testing for BBVs in custody suites is feasible and identifies a high proportion of individuals with active HCV infection, with the majority being commenced on antiviral treatment, including some that were previously difficult to engage. Viral Hepatitis services should consider expanding BBV testing to custody suites to help work towards HCV elimination.

## Author Contributions

Danielle Rayner: formal analysis, writing – original draft, data curation. Kate McQue: data curation, formal analysis, project administration, writing – review and editing. Francesca Mccullough, Caroline Allsop, Carolyn Miller and Jenna Bell: data curation, patient management, project administration, writing – reviewing and editing. Kerry Jones: data curation, methodology, project administration. Stuart McPherson: conceptualisation, visualisation, methodology, supervision, writing – review and editing, guarantor.

## Conflicts of Interest

SMc has received consultancy/speakers fees from Abbvie, Allergan, BMS, Gilead, Intercept, Magrigal, MSD, Novo Nordisk, Norgine, Novartis and Sequana. The other authors have no conflict to disclose.

## Data Availability

The data that support the findings of this study are available from the corresponding author upon reasonable request.
